# Characterization of health concerns in people with neurofibromatosis type 1

**DOI:** 10.1002/mgg3.2077

**Published:** 2022-11-28

**Authors:** Jane Fleming, Oliver Morgan, Claire Wong, Timothy E. Schlub, Yemima Berman

**Affiliations:** ^1^ Department of Clinical Genetics Northern Sydney Local Health District Sydney New South Wales Australia; ^2^ Faculty of Health and Medicine University of Sydney, Northern Clinical School Sydney New South Wales Australia; ^3^ Department of Clinical Genetics The Children's Hospital at Westmead Westmead New South Wales Australia; ^4^ Sydney School of Public Health, Faculty of Medicine and Health University of Sydney Camperdown New South Wales Australia

**Keywords:** cosmetic treatment, itch, neurofibromatosis, quality of life, surveillance

## Abstract

**Background:**

Neurofibromatosis 1 (NF1) is a common cancer predisposition syndrome. Affected individuals require lifelong surveillance and often suffer progressive disfigurement due to cutaneous neurofibromas. The aim of this research was to characterize health concerns and quality of life (QOL) in a population cohort.

**Methods:**

An online survey was completed by 68 adults and 32 parents of children with NF1, and 60 controls. The survey included the Skindex‐29 QOL scale, 5D‐itch scale, and additional health questions.

**Results:**

Frequency of itch was high in children (50%) and adults (69%), with most expressing interest in treatment for itch. The presence of itch and increased visibility of NF1 were predictors of poorer QoL. Many adults (53%) and parents (44%) desired access to treatment to improve cosmetic appearance. Muscle weakness/tiredness was also prevalent amongst (60–70%) adults and children with NF1. Two‐thirds of adults with NF1 reported limited awareness of NF1 services and poor knowledge of surveillance, particularly breast screening in young women.

**Conclusion:**

This study highlights the impact of NF1‐related itch and visibility in adults and children with a need for cosmetic and itch treatment. The findings emphasize a need for strategies to promote awareness, and access to management and treatment of NF1 in adults.

## Graphical abstract

NF1 is a common cancer predisposition syndrome that requires lifelong surveillance. The aim of this research was to characterize health concerns and QoL in a population cohort. This study highlights that the presence of itch and increased visibility of NF1 complications impacts the QoL of adults and children.

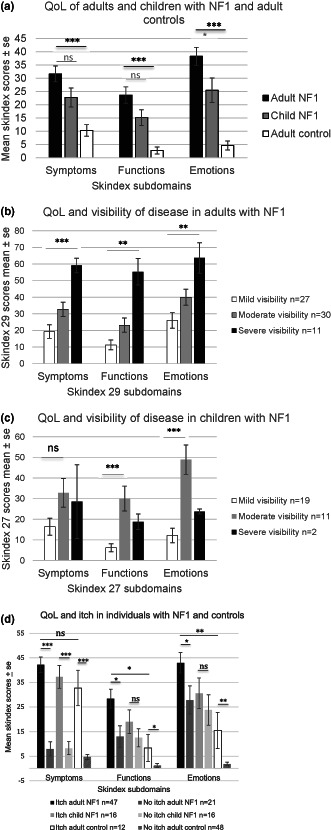

## 1 INTRODUCTION

Neurofibromatosis 1 (NF1) is a progressive, autosomal dominant, hereditary disorder with a birth incidence of approximately 1 in 2000 to 1 in 3000 live births (Evans et al., 2010; Lammert et al., 2005; Poyhonen et al., 2000; Uusitalo et al., 2015) and a prevalence of approximately 1 in 3000 to 1 in 5000 (Evans et al., 2010; Kallionpaa et al., 2018; Lammert et al., 2005; Poyhonen et al., 2000). The disease occurs due to a pathogenic variant in the NF1 gene (OMIM: 162200) that encodes the tumor suppressor neurofibromin (Theos & Korf, 2006). Individuals affected by NF1 have an increased susceptibility to the formation of benign and malignant tumors, particularly cutaneous, subcutaneous and plexiform neurofibromas; peripheral nerve sheath tumors; and optic gliomas. NF1 can also cause non‐tumor manifestations including scoliosis, vasculopathy, and neurocognitive disorders (Stewart et al.). The condition is most often diagnosed in childhood based on defined clinical criteria (Legius et al., 2021).

The clinical manifestations of NF1 can have a significant impact on cosmetic appearance (Dunning‐Davies & Parker, 2016), with benign cutaneous neurofibromas (cNFs) present in almost all adults (Fjermestad et al., 2018). cNFs increase in size and number with age, with associated itch, pain, and bleeding (Duong et al., 2011; Huson et al., 1988). The development of multiple cutaneous lesions can be significantly disfiguring and negatively impact health‐related and skin‐related quality of life (QoL) (Kodra et al., 2009; Maguiness et al., 2021). Indeed, individuals with NF1 have rated cutaneous manifestations of NF1 as most concerning despite the prospect of other potentially life‐threatening complications (Crawford et al., 2015).

Itch (Brenaut et al., 2016) is increasingly being recognized as a prominent feature of NF1. A French study of 40 people with NF1 found itch frequency to be daily or almost daily in up to 70% of participants (Brenaut et al., 2016). By comparison, a retrospective cohort study reported itch frequency as 19.4% and itch was a significant predictor of mortality in children (Khosrotehrani et al., 2005). Further support for itch as a clinically important symptom in NF1 has been highlighted by a number of case studies that found itch to be associated with a spinal cord tumor (Johnson et al., 2000) and brainstem gliomas (Darken et al., 2009). Despite these findings, there remains a paucity of data on the frequency, characteristics, and psychosocial impact of itch in NF1.

Recent reports suggest muscle weakness (Cornett et al., 2015; Stevenson et al., 2012) may be another under identified feature of NF1, that may be potentially treatable. Deficiencies in muscle function have been identified in NF1 patients, including decreased muscle size, muscle strength, and motor proficiency (Cornett et al., 2015; Stevenson et al., 2012; Vasiljevski et al., 2020). There is some evidence from mouse studies to suggest reduced muscle strength may be related to abnormal Ras or cAMP signaling (Sullivan et al., 2014). Furthermore, murine studies have demonstrated accumulation of intramyocellular lipids that resolve with carnitine supplementation (Vasiljevski et al., 2020). Recently, treatment of a small cohort of children with L‐carnitine led to a significant improvement in muscle function including a 6‐min walk test, and dynamometry (Vasiljevski, 2020).

Regular surveillance for individuals with NF1 is recommended to enable early detection of malignancy (including early breast screening for women with NF1) or other complications (Stewart et al., 2018). However, poor adherence to screening has been reported potentially due to cognitive impairments and the impact of multiple appointments. Indeed, it has been reported many children with NF1 are lost to follow up after transition to adult services and many only reconnect when planning a family (Crawford et al., 2016).

The primary aim of this survey‐based study was to evaluate QoL and characterize the prevalence and impact of health concerns such as itch and muscle weakness in a community sample of people living with NF1 and identify potential areas for improvement in NF care.

## 2 METHODS

### 2.1 Editorial policies and ethical considerations

Approval from the Hunter New England Human Research Ethics Committee (HNEHREC) (reference: 2019/ETH01229) to conduct this study was received. Participants provided tacit consent, implied through submission of the survey, as permitted by the HNEHREC. The study conforms to recognized standards such as the Declaration of Helsinki.

A cross‐sectional study was undertaken to examine the health concerns of people living with NF1. The primary outcome of the study was to identify characteristics of itch, muscle weakness, range of severity, visibility, and QoL within the Australian NF1 population in comparison to a control sample in addition to gaps in service delivery. Secondary outcomes included analysis of factors that might improve health outcomes in this population.

### 2.2 Instrumentation

An online survey (titled: Health concerns in Neurofibromatosis Type 1) consisting of 115 items was constructed by the research team. Survey items were derived from the 5D itch scale (Elman et al., 2010), Riccardi Severity Scale (Riccardi & Kleiner, 1977), Ablon Visibility Scale (Ablon, 1996), and Skindex‐29, a skin‐disease‐specific QoL questionnaire (Chren, 2012). Additional questions relating to itch characteristics, muscle weakness, breast cancer‐screening awareness, and access to services for NF1 were also included.

### 2.3 Modified Riccardi Severity Scale

The Riccardi Scale categorizes NF1 into four different grades of severity based on the impact of NF1 on health and well‐being (Riccardi & Kleiner, 1977). For this study the scale was modified from a clinician reported outcome, to a patient self‐reported outcome, consistent with adaptations made by Page (Page et al., 2006).

### 2.4 Modified Ablon Scale

The Ablon Scale measures three different grades of visibility based on the patient's appearance while fully clothed (Ablon, 1996). Grade 1 indicates no visible NF1 manifestations, grade 2 some visible NF1 manifestations, and grade 3 obvious visible NF1 manifestations. Again, the scale was modified from a clinician reported outcome, to a participant self‐reported outcome, consistent with adaptations made by Page (Page et al., 2006).

### 2.5 Skindex‐29 skin related QoL scale

Skindex‐29 is a validated instrument consisting of 30 items that measures the impact of skin disease on three aspects of QoL: functioning, physical symptoms, and emotions (Chren, 2012). [*Skindex 29 contact information and permission to use: Mapi Research Trust*, *Lyon*, *France*, https://eprovide.mapi‐trust.org]. Scores for each subdomain range from 0 (no effect) to 100 (effect experienced continuously). A pediatric adaptation, the pSkindex‐27, has been validated for use in cutaneous lupus erythematosus disease which removed two items relating to sex life and affection difficulty (AlE'ed et al., 2018). These questions were similarly excluded in the analysis of surveys completed for children with NF1 in this study.

### 2.6 Modified 5D itch scale

The 5D itch scale is a 23‐item validated instrument used to measure five domains: of chronic itch: duration, degree, direction, disability, and distribution (Elman et al., 2010). Scores range from 5 to 25, with higher scores indicating a higher severity of chronic itch. The scale was modified for readability to account for cognitive difficulties associated with NF1 (Payne et al., 2010). For example: “rate the impact of your itching on the following activities” was changed to “how often does itch affect these activities”.

### 2.7 Participants

Individuals with a clinical diagnosis of NF1 were invited to participate in an online survey via advertisements in newsletters and social media groups of Australian support groups: Children's Tumour Foundation (CTF), Genetic Alliance Australia, the Genetic Support Network Victoria, Rare Voices, Syndromes Without a Name Australia, and the Genetic and Rare Disease Network. Respondents were required to be adults with NF1, or caregivers of children with NF1 (<18 years).

Participants without a diagnosis of NF1 were invited to participate as a control group via the CTF closed Facebook group, the Royal North Shore Hospital newsletter, and the University of Sydney's online Research Volunteer portal.

Recruitment for the study occurred 14 June 2019–28 February 2020. No identifying information was collected.

Study data were collected using Research Electronic Data Capture (REDCap) hosted at Royal North Shore Hospital (Harris et al., 2009).

### 2.8 Statistical considerations

Analysis of variance (ANOVA), independent‐samples *t*‐tests, Mann–Whitney U, Pearson Chi‐square tests, Spearman's rank‐order correlations, and multiple linear regression tests were conducted using SPSS (IBM Corp. Released 2017. IBM SPSS Statistics for Windows, Version 25.0. Armonk, NY: IBM Corp). *p*‐values of <0.05 were considered statistically significant. Values are reported as frequencies (%), or means ± *SD*, unless otherwise stated. Adult ages were grouped into 18–29, 30–39, 40–49, 50–59 and 60 years and children's ages were grouped into ≤10 years and >10 years above for QoL analyses. A sample size of 128 adults, based on 64 participants in each group (individuals with NF1 and controls—the approximate numbers in this study) was calculated to have 80% power to detect an effect size of at least 0.5 for QoL and itch scores at a 5% significance level.

## 3 RESULTS

A total of 165 surveys were fully completed and accepted for analysis, with a further 41 surveys excluded as they were incomplete. Five additional surveys were omitted as they were completed either by respondents outside Australia, or it was unclear if they were completed by an adult or for a child with NF1. The final sample consisted of 68 adults with NF1, 32 children with NF1 (surveys completed by their parents), and 60 adult individuals without NF1 who acted as the control group. The majority of respondents in the NF1 group and control group were female and approximately half of the children with NF1 were female. Over half of NF1 respondents self‐reported itch symptoms compared to just under one in five of controls. In terms of NF1 severity most adults with NF1 scored in the mild to moderate range (grade 2) and most parents of children with NF1 reported a higher degree of severity, scored as grade 3. The majority of adults with NF1 reported grade 2 visibility whereas children with NF1 had a reduced median visibility score of grade 1 (Table 1).

**TABLE 1:** Demographics

	Adults with NF1 (*n* = 68)	Children with NF1 (*n* = 32)	Control (*n* = 60)
Age mean + *SD*: range	42.17 ± 12.7 (18–68 years)	9.62 ± 4.9 (2–17 years)	43.30 ± 13.8 (18–80 years)
Gender			
Female	53 (78%)	17 (53%)	52 (87%)
Male	15 (22%)	15 (47%)	8 (13%)
State/Territory			
NSW	29 (43%)	11 (34%)	46 (77%)
VIC	17 (26%)	7 (22%)	4 (7%)
QLD	7 (10%)	6 (19%)	5 (8%)
WA/SA/ACT/TAS/NT	15 (22%)	8 (25%)	5 (8%)
Geographic area			
Metropolitan	39 (57%)	16 (50%)	50 (83%)
Rural	29 (41%)	16 (50%)	10 (17%)
*Self‐reported muscle symptoms*			
Muscle weakness			
Yes	43 (63%)	24 (75%)	6 (10%)
Muscle tiredness			
Yes	43 (63%)	23 (72%)	9 (15%)
Self‐reported itch symptoms			
Yes	47 (69%)	16 (50%)	12 (20%)
NF1 self‐rated severity (modified Riccardi scale)			
Grade 1	5 (7%)	6 (19%)	–
Grade 2	34 (50%)	6 (19%)	–
Grade 3	17 (25%)	13 (40%)	–
Grade 4	12 (18%)	7 (22%)	–
Mean severity ± *SE*	2.5 ± 0.1	2.7 ± 0.18	
Mean severity gender	M 2.67: F 2.49	M 2.61: F 2.71	
Median severity	2	3	
Gender versus Severity^^^	*z* = −0.496 *p* = 0.69	*z* = 0.358 *p* = 0.72	
Age versus severity^&^	*F* = 3.004 *p* = 0.037	*F* = 3.70 *p* = 0.023	
NF1 self‐rated visibility (modified Ablon Scale)			
Grade 1	18 (39%)	8 (59%)	–
Grade 2	30 (44%)	11 (34%)	–
Grade 3	11 (16%)	2 (6%)	–
Mean visibility ± *SE*	1.76 ± 0.09	2.18 ± 0.18	
Mean visibility gender	M 1.73: F 1.77	M 1.43: F 1.50	
Median visibility	2	1	
Gender versus visibility^^^	*z* = 0.289 *p* = 0.77	*z* = −0.044 *p* = 0.97	
Age versus visibility	*F* = 3.231 *p* = 0.046	*F* = 4.52 *p* = 0.020	

*Note*: Male (M), female (F). ^ Mann–Whitney U test, differences between male and female severity scores, & ANOVA comparison of age and visibility scores.

In adults and children with NF1, no significant difference in severity or visibility scores was identified between males and females. However, increased age was significantly associated with increased severity and increased visibility (Table 1). Multiple linear regression analysis demonstrated that for adults and children with NF1 increased age remained a predictor of increased visibility but not gender of the individual with NF (adults with NF1 standardized coefficient beta age = 0.293, *p* = 0.017, gender = 0.055, *p* = 0.649: children with NF1: age = 0.391, *p* = 0.043; gender = 0.196, *p* = 0.297). For adults and children with NF1, increased age also remained a predictor of increased severity but not gender: (adults with NF1 age, *R* ^2^ = 0.066, standardized coefficient beta age = 0.244 *p* = 0.047, gender = 0.058, *p* = 0.629; children with NF1 age *R* ^2^ = 0.137, standardized coefficient beta age = 0.79, *p* = 0.011, gender = 0.119, *p* = 0.506).

### 3.1 Muscle weakness

**FIGURE 1:**
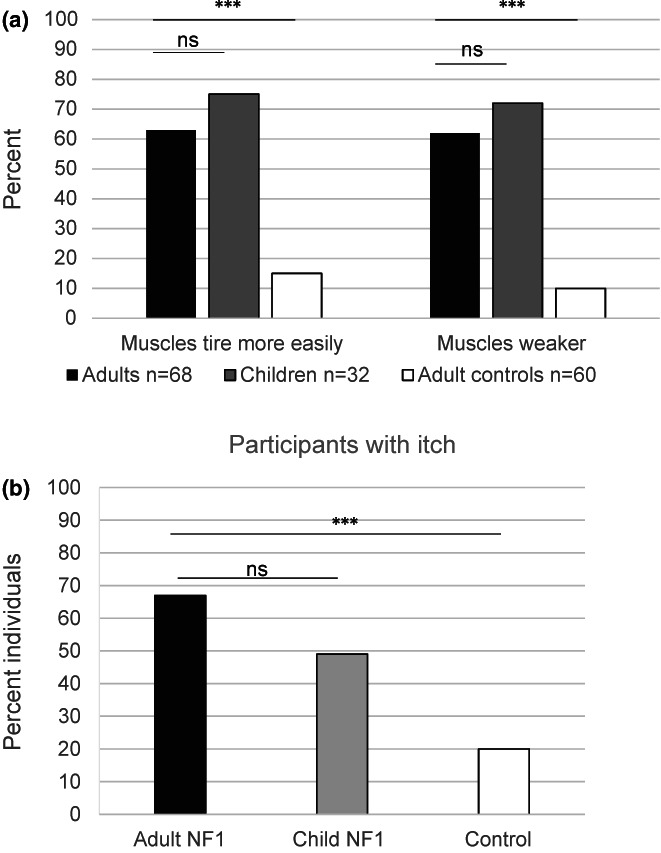
Health concerns of adults and children with NF1, and an adult control sample with (a). muscle weakness and muscle tiredness associated with neurofibromatosis type 1 and (b). self‐reported itch associated with neurofibromatosis type 1. Significance *p < 0.05, **p < 0.01, ***p < 0.001, ns = not significant.

A significantly increased proportion of adults with NF1 self‐reported muscle weakness and muscle tiredness compared to adult controls (muscle weakness mean difference = 0.503, *p* < 0.001; muscle tiredness: mean difference = 0.453, *p* < 0.001). Similarly, parents reported muscle weakness and tiredness in children with NF1 that were not significantly different to that in adults with NF1 (muscle weakness mean difference = 0.147, *p* = 0.153; muscle tiredness mean difference = 0.116, *p* = 0.265) (Figure 1a). There was no association between muscle weakness or muscle tiredness and gender in adults (muscle weakness c^2^ 0.001, 1, *p* = 0.979; muscle tiredness c^2^ 1.49, 1, *p* = 0.222) or children (muscle weakness Fisher *p* = 1.000; muscle tiredness Fisher *p* = 1.000) and no association between muscle weakness or muscle tiredness and age for adults (muscle weakness mean difference = 1.050, *p* = 0.735; muscle tiredness mean difference = 0.670 *p* = 0.829) or children (muscle weakness mean difference = 1.729, *p* = 0.329; muscle tiredness mean difference 1.708, *p* = 0.317).

### 3.2 Itch

Adults with NF1 reported significantly more itch than a control sample of adults with itch (mean difference = 0.491, *p* < 0.001) (Figure 1b). Although more adults with NF1 reported itch in comparison to children with NF1 (mean difference = 0.191, *p* = 0.066) this did not reach significance.

Respondents who reported symptoms of itch in the past two weeks completed the 5D itch scale, with adults with NF1 (*n* = 47/68; mean 13.19 ± 3.28 [range 7–19]) and children with NF1 (*n* = 16/32; mean 12.56 ± 3.27 [range 9–20]). A significantly smaller number of controls reported itch, with similar severity (*n* = 12/60; mean 10.75 ± 2.92 [range 6–15]) (*z* = −2.19 *p* = 0.029). These 5D itch scores represent moderate severity, comparable to burns patients (Elman et al., 2010) (Table 2). Most respondents with NF1 and itch reported experiencing itching for less than six hours a day, their itching was of a mild to moderate intensity with over a third of respondents reported itching on 3–5 parts of the body. For most respondents, itch was reported to occur almost daily to everyday. Generally, the itching intensity had not changed during the preceding two weeks. For two thirds of adults and children with NF1, itch “sometimes”, or more frequently, impacted falling asleep with other activities also impacted. Almost two thirds of adults with NF1 (62%) also reported that itch had an emotional impact (sometimes or more frequently) as compared to significantly fewer controls (25%) (c^2^ = 5.19, df = 1, *p* = 0.023) (Table 2).

**TABLE 2:** Characteristics of itch as reported over the previous 14 days: A comparison between individuals with NF1 and controls

Characteristics of itch	Adult + child NF1 (*n* = 63)	Adult NF1 (*n* = 47)	Child NF1 (*n* = 16)	Control (*n* = 12)
Frequency				
None	1 (2%)	1 (2%)	0	0
<1/week	8 (13%)	6 (13%)	2 (12)	4 (33%)
Every week	18 (29%)	13 (28%)	5 (31)	5 (42%)
Most days	24 (38%)	21 (44%)	3 (19%)	2 (17%)
Everyday	12 (19%)	6 (13%)	6 (38%)	1 (8%)
Duration				
<6 h	46 (73%)	33 (70%)	13 (81%)	11 (92%)
6–12 h	5 (8%)	5 (11%)	0	1 (8%)
12–18 h	4 (6%)	4 (8%)	0	0
18–23 h	1 (2%)	0	1 (6%)	0
All day	7 (11%)	5 (11%)	2 (13%)	0
Degree				
Not present	3 (%)	3 (6%)	0	0
Mild	26 (%)	18 (38%)	8 (50%)	8 (67%)
Moderate	26 (%)	20 (43%)	6 (38%)	4 (33%)
Severe	7 (%)	5 (11%)	2 (12%)	0
Unbearable	1 (2%)	1 (2%)	0	0
Direction				
Gone	2 (3%)	1 (2%)	1 (6%)	2 (17%)
Better still there	4 (6%)	3 (6%)	1 (6%)	0
A little better	2 (2%)	0	2 (12.5%)	0
No change	50 (79%)	40 (85%)	10 (63%)	9 (75%)
Itch worse	5 (8%)	3 (6%)	2 (12.5%)	1 (8%)
Distribution				
0–2 body parts	15 (24%)	10 (21%)	5 (31%)	4 (33%)
3–5 body parts	23 (37%)	16 (34%)	7 (44%)	6 (50%)
6–10 body parts	16 (25%)	12 (26%)	4 (25%)	1 (8%)
11–13 body parts	5 (8%)	5 (11%)	0	1 (8%)
14–16 body parts	4 (6%)	4 (8%)	0	0
Disability impact on sleep				
Never	18 (29%)	14 (30%)	4 (25%)	8 (67%)
Sometimes falling asleep	23 (37%)	17 (36%)	7 (44%)	3 (25%)
Often falling asleep	8 (13%)	5 (11%)	3 (19%)	0
Sometimes falling asleep and wakes me	10 (16%)	9 (19%)	1 (6%)	1 (8%)
Often falling asleep and wakes me	3 (5%)	2 (4%)	1 (6%)	0
Itch location (NFs)				
On NFs only	7 (11%)	6 (12.8%)	1 (6%)	–
On NFs and other areas of the skin	41 (65%)	34 (72.3%)	7 (44%)	–
Not on NFs	15 (23%)	7 (14.9%)	8 (50%)	–
Itch location (body)				
All over the body	30 (48%)	25 (53.2%)	5 (31%)	1 (8%)
Certain parts of the body only	33 (52%)	22 (48.8%)	11 (69%)	11 (92%)
Triggers				
When new NFs appear	15 (24%)	12 (26%)	3 (19%))	–
When NFs grow bigger	11 (18%)	8 (17%)	3 (19%)	–
Heat	36 (57%)	27 (57%)	9 (56%)	–
Physical activity	15 (24%)	10 (21%)	5 (31%)	–
Time of day (e.g., evening)	15 (24%)	9 (19%)	6 (38%)	–
During pregnancy (ladies)	12 (19%)	11 (23%)	1 (6%)	–
No specific triggers	27 (43%)	23 (49%)	4 (25%)	–
Emotions				
Never affect	11 (18%)	8 (17%)	3 (19%)	3 (25%)
Rarely affects	14 (22%)	10 (21%)	4 (25%)	6 (50%)
Sometimes affects	23 (37%)	19 (40%)	4 (25%)	2 (17%)
Often affects	10 (16%)	6 (13%)	4 (25%)	1 (8%)
Always affects	5 (8%)	4 (9%)	1 (6%)	0 (0%)

*Note*: Itch frequency, duration, degree, direction, distribution as reported over the previous 14 days. 5D itch score calculated from duration, degree, direction, distribution, and disability.

Common triggers and characteristics of itch were also identified, with itch being triggered by development of new cNFs in adults (26%) and children (19%) with NF1, and heat being the most common trigger across both adults (57%) and children (56%). Over a third of adults with NF1 and itch (38.3%) also reported scratching (in free text responses) compared to 25% of controls; 21% also mentioned bleeding secondary to scratching as a concern (compared to 0% of controls).

Most adults 35/47 (74%), and parents of children 14/16 (88%) with NF1 who had itch expressed an interest in trying medications that might reduce their itch. Many adults with NF1 reported trying steroid creams (19/47 [40%]), emollients (29/47 (62%)), antihistamines (27/47 (57%)) and other treatments (Doxepin (1/47) (a tricyclic antidepressant); Depran (1/47) (a selective serotonin reuptake inhibitor); psoriasis cream (1/47) and over the counter bath and shower products (4/47). Less than half of adults with NF1 reported treatment improved itch “somewhat” or “a lot” with the following treatments: steroid creams (5/19 [26%]), emollients (4/19 [21%]), antihistamines (adults 7/27 [26%]) and other (3/7 [43%]). Parents/caregivers reported similar results for children with NF1, who had tried steroid creams, emollients, and antihistamines. Side‐effects from treatment included: drowsiness (Depran); emotional ups and downs (Doxepin); weight gain, constipation, an odd feeling (unspecified oral medication); and greasy skin (steroid creams).

### 3.3 Skin‐related quality of life

**FIGURE 2:**
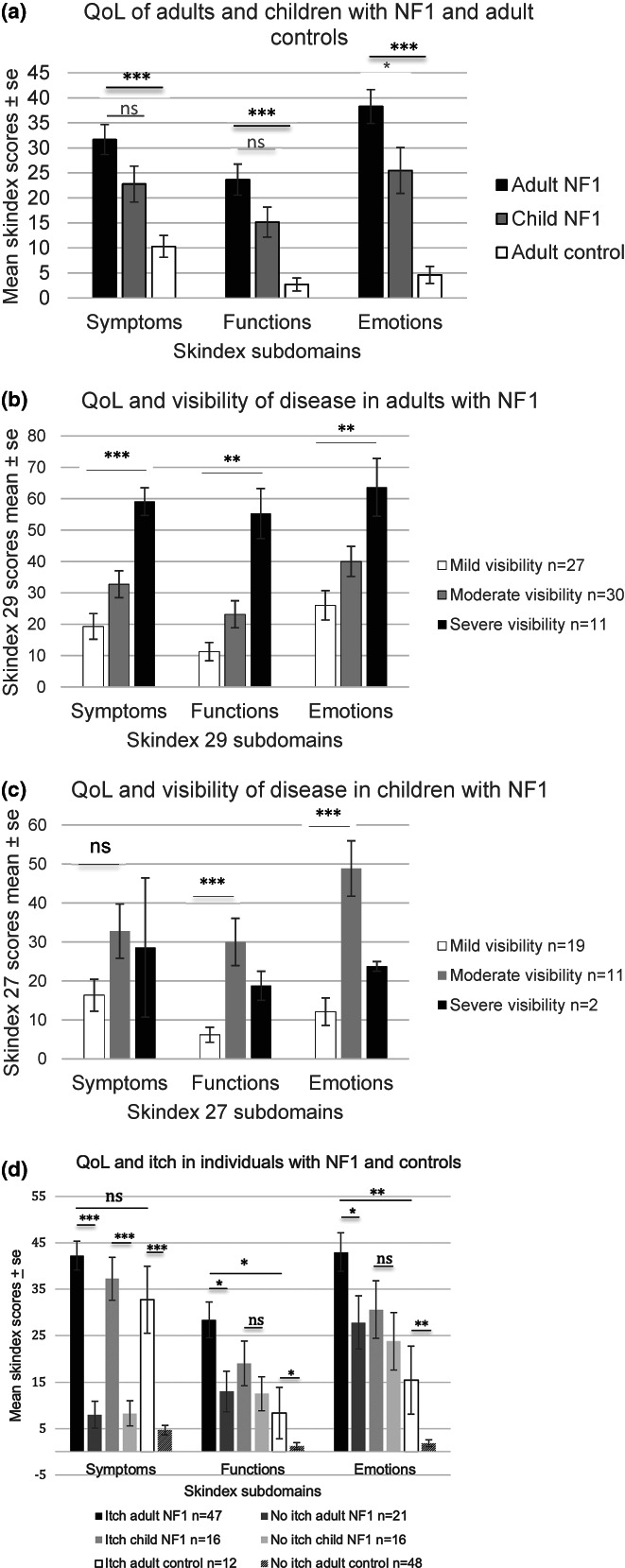
Skin‐related quality of life (QoL) scores presented for (a). adults with NF1, children with NF1 and an adult control sample. (b) adults with increasing visibility of NF1, Ablon: grade 1 (mild visibility) to grade 3 (obviously visible NF1 manifestations). (c) children with increasing visibility of NF1, Ablon: grade 1 (mild visibility) to grade 2 (moderate visibility NF1) (d). adults and children with NF1 and an adult control sample with and without itch. Statistical analysis is between adults with NF1 and adult controls. Significance *p < 0.05, **p < 0.01, ***p < 0.001, ns = not significant.

Skin‐related QoL scores were increased in adults with NF1, indicating poorer QoL, especially in the emotions subdomain when compared to adult control values (physical symptoms mean difference = 21.37, *p* < 0.001; functioning, mean difference = 20.98, *p* < 0.001; emotions, mean difference = 33.69, *p* < 0.001). Similar results were observed for children with NF1 using the modified Skindex 27 measure; however, adults had significantly poorer QoL scores than children in the emotions subdomain (physical symptoms mean difference = 8.90, *p* = 0.086; functioning mean difference = 8.50, *p* = 0.095; emotions mean difference = 12.08, *p* = 0.033) (see Figure 2a). This decrease in skin‐related QoL in adults with NF1 was not significantly associated with gender (physical symptoms mean difference = 0.478, *p* = 0.947; functioning mean difference = 13.134, *p* = 0.082; emotions mean difference = 8.418, *p* = 0.322) or age (physical symptoms *p* = 0.065; functioning *p* = 0.135; emotions *p* = 0.189). However, in children with NF1 although gender was not associated with poorer QoL (physical symptoms mean difference = 4.365, *p* = 0.555; functioning mean difference = 2.794, *p* = 0.635; emotions mean difference = 3.929, *p* = 0.665) increased age (>10 years) was associated with poorer QoL in the functioning and emotions subdomains (physical symptoms mean difference = 9.921, *p* = 0.174, functioning mean difference = 11.524, *p* = 0.043; emotions mean difference = 19.802, *p* = 0.023). In comparison to potentially clinically relevant cut offs of impaired skin‐related QoL (Skindex 29) (Prinsen 2011) our results suggest that approximately 38%, 44%, and 65% of adults with NF1 have at least mildly impaired QoL in the “physical symptoms” (cut off ≥ 39), “functioning” (cut off ≥ 21) and “emotions” (cut off ≥ 24) subdomains, respectively.

Respondents with more visible NF1 reported significantly greater impact on all three QoL domains (ANOVA adults with NF1: physical symptoms mean difference = 36.77 *p* < 0.001; functioning mean difference = 44.04, *p* ≤ 0.001; emotions mean difference = 37.62, *p* = 0.001 between grade 1 and grade 3 visibility). Comparable results were evident in children with NF1, although this was not significant for the physical symptoms subdomain nor for those with grade 3 visibility—likely due to insufficient numbers in this group (ANOVA children with NF1: physical symptoms mean difference = 15.63, *p* = 0.102; functioning mean difference = 21.79, *p* < 0.001; emotions mean difference = 36.76, *p* < 0.001) [mean differences grade 1 and 2] (Figure 2b,c). Respondents with more severe NF1 reported significant impacts on the physical symptoms' subdomain of Skindex‐29 (ANOVA adults with NF1: physical symptoms mean difference = 34.52, *p* = 0.034; functioning mean difference = 20.97, *p* = 0.163; emotions mean difference = 6.75, *p* = 0.382 [mean differences grade 1 and 4].

Adults with NF1 and itch also reported significantly more impact on all three QoL domains (physical symptoms mean difference = 32.92, *p* < 0.001; functioning mean difference = 15.42, *p* = 0.022; emotions mean difference = 15.42, *p* = 0.043) than adults with NF1 without itch (see Figure 2d). Itch also had a similar impact on the “physical symptoms” subdomain in children with NF1 (children: physical symptoms: mean difference = 26.95, *p* < 0.001; functioning mean difference = 6.64, *p* = 0.251; emotions mean difference = 8.74, *p* = 0.247) compared to children with NF1 without itch; and the “physical symptoms”, “functioning” and “emotions” subdomains in adult controls with itch (physical symptoms: adult controls: mean difference = 26.82, *p* < 0.001; functioning: adult controls: mean difference = 7.08, *p* = 0.024; emotions: adult controls: mean difference = 13.54, *p* = 0.001) when compared to controls without itch (Figure 2d).

To determine the relationship between 5D itch and skin‐related QoL scores (Skindex 29), a Spearman's rank‐order correlation was performed. The strongest relationship between 5D itch and QoL was in the “physical symptoms” subdomain (*r*_*s*_ = 0.742, *n* = 47, *p* < 0.01). There was a moderately positive correlation between the “functioning” subdomain and 5D itch score (*r*_*s*_ = 0.439, *n* = 47, *p* < 0.01) and a weak positive correlation, between the “emotions” subdomain (*r*_*s*_ = 0.346, *n* = 47, *p* < 0.05). For individual items on the Skindex 29 QoL measure, 41% of respondents reported their skin itched (physical symptoms subdomain) often or all the time, and over a third felt “frustrated” (38.3%) “annoyed” (36.8%) or “worried their skin condition may get worse” (35.3%) often or all the time (emotions subdomain).

Multiple linear regression to assess the contribution of each of the variables: itch, severity, and visibility in adults with NF1 (*n* = 68) demonstrates that in this study only visibility was independently associated with all aspects of QoL (physical symptoms *R* ^2^ = 0.522, standardized beta coefficient = 0.296 (*SE* = 3.28), *p* = 0.003; functioning: *R* ^2^ = 0.317, standardized beta coefficient = 0.501 (*SE* = 4.17), *p* ≤ 0.001; and emotions: *R* ^2^ = 0.208; standardized beta coefficient = 0.407 (*SE* = 5.01), *p* = 0.002); with visibility accounting for 7%, 20% and 13% of the variance in each of the three subdomains, respectively. However, itch makes the largest contribution in the “physical symptoms” subdomain symptoms *R* ^2^ = 0.522, (itch standardized coefficient beta 0.522, [*SE* = 4.75], *p* < 0.001), accounting for 25% of the variance.

### 3.4 Surveillance

**FIGURE 3:**
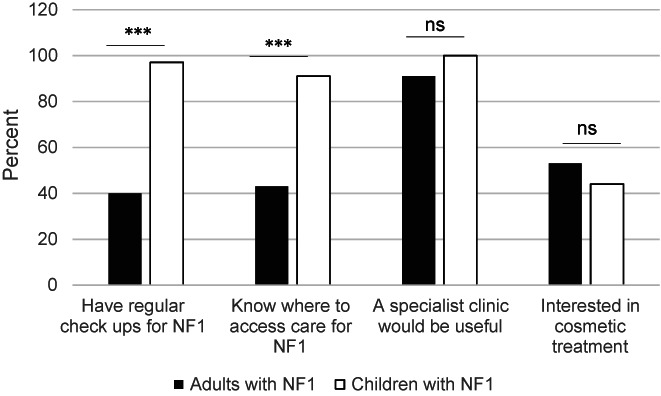
Surveillance in adults and children with NF1 and interest in specialist care. Comparison of cohorts of adults and children with NF1. Significance *p < 0.05, **p < 0.01, ***p < 0.001, ns = not significant.

Although the majority of children with NF1 were reported to have regular surveillance (annual screening recommended for children [Stewart et al., 2018]); less than half of adults with NF1 reported having regular check‐ups (mean difference = 0.572, *p* < 0.001) (Figure 3). In addition, most parents/caregivers knew where to access care for their children with NF1, whereas only 43% of adults with NF1 knew where to access care for themselves (mean difference = 0.480, *p* < 0.001) (Figure 3). For adults with NF1 knowing where to access care (c2 = 1.703, df = 1, *p* = 0.192) and having check‐ups (c2 = 0.554, df = 1 *p* = 0.457) was not associated with living in an urban location. Of interest, a third of adults with NF1 (10/29) who knew where to access care for NF1, did not have regular health check‐ups.

At the time of this study, breast cancer screening for women with NF1 in Australia was recommended annually from the age of 35 years, compared to 50 years of age for general population screening (Cancer Institute NSW, 2021). For women with NF1 (mean age 43.06 ± 13.6 years), 21/34 (62%) over 35 years reported having regular mammograms. However, of the 13 women over 35 years not undertaking regular screening, eight were unaware of when to start screening; and five women intended to start screening at 40 or 50 years of age. Of the women with NF1 aged <35 years, only 3/17 (18%) intended to start screening at 35 years in line with recommendations current at the time of the survey (none were 18 to 29‐years). Another two women (32 and 34 years) reported having breast screening early (reasons unascertained). A further 14/17 (82%) women were intending to start regular screening at ages that were not in line with recommendations (Cancer Institute NSW, 2021), or they were unaware of when to start screening (*n* = 7).

In comparison, 14/16 (87.5%) women in the control group aged over 50 years, reported having regular screening, with 18/34 (64%) women under 50 years intending to start screening at 50 years (4 were18‐29 years). Four women under 50 years (35–49 years) reported having breast screening early (reasons unascertained). Only 6/34 (18%) of women who had not had screening in the control group were unaware of when to start screening; significantly fewer than the 14/30 women (45%) in the NF1 population (mean difference = 0.290, *p* = 0.12).

### 3.5 Treatment of NF1

Most adults with NF1 and all parents of children with NF1 felt a specialist NF clinic would be useful. In addition, almost half of adults with NF1 and parents with children with NF1 were interested in cosmetic treatments for skin lesions (Figure 3). However, half of these adults (21/36 [58%]) and children with NF1 (7/14 (50%)) did not have someone who treated their skin. Barriers to accessing cosmetic treatment for adults and children respectively included: distance to travel (9/36 (25%); 2/14 (14%)), cost (8/36 (22%); 1/14 (7%)), taking time off work or school (4/36 (11%); 2/14 (14%)), and discomfort or pain from treatment (2/36 (6%); 4/14 (28%)).

## 4 DISCUSSION

The results of this study provide evidence of potentially treatable health concerns of importance to adults and children with NF1 that in many cases have been under recognized in the NF1 population.

A significant proportion of adults and children with NF1 in this population sample exhibited muscle weakness and tiredness, itch, and worries regarding cosmetic appearance. Two thirds of adults and three quarters of children (by parental report) reported symptoms of muscle weakness and muscle tiredness. This is consistent with previous studies that have shown muscle deficits in individuals with NF1 (Cornett et al., 2015; Stevenson et al., 2012; Vassallo et al., 2020). The high prevalence of muscle weakness and fatigue and promise of a safe dietary supplement (L‐Carnitine) that may improve muscle function, support the need for further studies to investigate interventions that may improve muscle function in people with NF1.

Itch was reported by 69% of adults with NF1 and almost 50% of children. The adult frequency is similar to the proportion (70%) reported in a small study by Brenault and colleagues (Brenaut et al., 2016). Another larger, but retrospective study of patient records reported a lower frequency of itch (19.4%) in adults and children with NF1 (Khosrotehrani et al., 2005). More recently, 14% of participants in a European community cohort described itch as being “most bothersome” (Guiraud et al., 2019). In a Norwegian non‐clinical cohort 22% felt itch had a “major impact” (Fjermestad et al., 2018).

The severity of itch as measured by the 5D itch score was in the moderate range of severity, consistent to that seen in the Brenault study, and similar to the 5D itch scores reported for burns patients (Elman et al., 2010). For two thirds of adults and children with NF1, itch impacted activities of daily living including falling asleep, work or schooling and leisure. Most adults (74%) and parents (88%) reporting itch expressed interest in medications to treat these symptoms. There are currently no established guidelines for the treatment of itch in NF1, exemplifying an area of need in NF1 that has yet to be addressed. The patterns of onset and triggers of itch identified in our study, where one third of patients found itch started with development of new neurofibromas, followed by secondary generalizations of interest in understanding both the natural history of NF1 and the pathophysiology of itch (Blakeley et al., 2018; Ortonne et al., 2018).

Visibility was an independent risk factor for poor quality of life across all subdomains (Skindex 29). Poorer skin‐related QoL was also highly impacted by itch. Although previous dermatological studies have clearly shown that chronic itch impairs QoL, to our knowledge this is the first study to investigate the impacts of NF1‐associated itch on QoL in adults. Our findings are supported by a study of children with NF1, where itch bother was found to be a predictor of health‐related QoL (Varni et al., 2019). Previous studies have also found poorer skin‐related quality of life in individuals with NF1, especially in the emotional subdomain (Brenaut et al., 2016; Kodra et al., 2009; Wolkenstein et al., 2001). Global NF1 severity and visibility scores reported in this study were similar to previous studies of non‐clinical (Page et al., 2006) and clinical cohorts of individuals with NF1 (Kodra et al., 2009; Wolkenstein et al., 2001); with severe and more visible disease associated with increased age.

Given the impact of visibility on skin‐related QoL, it is unsurprising that many adults and parents of children with NF1 in this study would like access to cosmetic treatments for NF1. Indeed, Cannon et al reported 48% to 58% of individuals with cNF would be willing to try experimental treatment, where most perceived a reduction of 33%–66% in number or size of cNFs would represent a meaningful response (Cannon et al., 2021). Currently there are a range of surgical cosmetic treatment options to reduce cNF burden (excision, electro‐dessication and laser (Erbium/YAG, CO_2_) (Chamseddin et al., 2019; Guiraud et al., 2019; Kim et al., 2013; Verma et al., 2018). However, laser may be only accessible through specialized clinics or private practices. Although many individuals are aware of surgical options, it has also been reported most would favor topical or oral medications (Cannon et al., 2021). Topical and oral medical treatments with MEK inhibitors and other agents also hold promise (Dombi et al., 2013; Koenig et al., 2012; Slopis et al., 2018), with a number of clinical trials pending. Ketotifen (a mast cell stabilizer) has also been reported to reduce itch and cNFs in NF1 (Riccardi, 1993); however, further studies into the effectiveness of this treatment are needed. Importantly, significant improvements in QoL have been reported when improved physical appearance, pruritis, discomfort and pain has been achieved through treatment of cNFs (Chamseddin & Le, 2020; Guiraud et al., 2019; Kim et al., 2013; Méni et al., 2015; Verma et al., 2018). Barriers to cosmetic treatments identified in this study, including cost and taking time off work, were similar to those reported previously, with many unwilling to tolerate side effects (e.g., pain, discomfort, nausea/vomiting) (Cannon et al., 2021).

Adults with NF1 in this cohort demonstrated poor attendance and limited awareness of NF services, similar to two previous Australian studies (Crawford et al., 2016; Oates et al., 2013). Given young adults are at highest risk for malignancy due to NF1 (Uusitalo et al., 2015) it is important that individuals with NF1 are effectively transitioned from pediatric to adult services, with easy access to regular surveillance, and the resources to seek specialist care when needed. Significantly, no difference in access or attendance at NF services was identified between individuals located in a rural setting, despite rural clients often being further from specialized NF services.

Awareness of breast screening guidelines for young women with NF1 (35–50 years) was also limited: therefore, there is a need for continued education and promotion to raise awareness of screening for this population. Indeed, our findings demonstrate that women with NF1 are less likely than the general population to undertake breast cancer screening at the recommended age, with younger women less knowledgeable about when to start surveillance. However, awareness of, and uptake of breast cancer screening through our clinic has demonstrated women who are seen regularly are keen to pursue surveillance, with 78% opting for annual breast screening (Crook et al., 2021). Provision of annual breast cancer surveillance for young women with NF1 (30–50 years) has also achieved high enrolment rates in a Canadian research study. These findings suggest young women with NF1 will engage in early breast screening programs when offered (Crook et al., 2021; Maani et al., 2019). Importantly, women (30–50 years) with NF1 did not exhibit increased psychological impact as a result of screening (Crook et al., 2021).

This study has a number of limitations that may be associated with some selection bias. There was a small sample size, which may not be representative of the NF1 population in Australia. In addition, the majority of participants were female; the data on children with NF1 was based on parental reports; only adult controls were recruited; and recruitment was through support groups. Although, NF1 diagnosis was not ascertained, the majority of adults and children recruited into the study were members of the National NF support group in Australia, which provides comprehensive information on NF. They were, therefore, likely well‐informed about NF1 and aware of their diagnostic status.

This study has implications for clinical practice in NF1 as health concerns regarding itch, cosmetic burden and muscle weakness associated with NF1 may be under‐reported; but can significantly impair QoL. In addition, cosmetic and drug therapies for the treatment of skin‐related manifestations of NF1 should be offered where available.

Future research could include (1) larger studies to explore the prevalence and impacts of the health concerns identified here; and (2) an exploration of NF community and clinician‐perceived barriers and possible strategies to promote access and awareness of NF services, screening recommendations, and improved service delivery to address the needs of individuals with NF1.

## 5 CONCLUSION

This study supports the incorporation of considerations of itch, muscle weakness, and cosmetic appearance in all NF1 consultations given the high prevalence of these concerns in adults and children, their impact on quality of life and wellbeing, and the potential for treatment. We also identified a significant and concerning need for improved awareness and access to adult NF1 services and surveillance (including breast screening).

## CONFLICT OF INTEREST

None of the authors has any conflict of interest to declare.

## AUTHOR CONTRIBUTIONS

Jane Fleming, Oliver Morgan, Claire Wong, and Yemima Berman made substantial contributions to the conception and design of the study, and obtained the clinical data. Timothy Schlub analyzed the data. All authors interpreted the data, and critically reviewed and approved the final manuscript. All authors agreed to be accountable for all aspects of the work in ensuring that questions related to the accuracy or integrity of any part of the work are appropriately investigated and resolved.

## ETHICS STATEMENT

The conduct of this study was approved by the Hunter New England Human Research Ethics Committee (HNEHREC) (reference: 2019/ETH01229). Participants provided tacit consent through submission of a survey.

## Data Availability

The data that support the findings of this study are available from the corresponding author upon reasonable request.
